# Understanding How CNNs Recognize Facial Expressions: A Case Study with LIME and CEM

**DOI:** 10.3390/s23010131

**Published:** 2022-12-23

**Authors:** Guillermo del Castillo Torres, Maria Francesca Roig-Maimó, Miquel Mascaró-Oliver, Esperança Amengual-Alcover, Ramon Mas-Sansó

**Affiliations:** Department of Mathematics and Computer Science, University of the Balearic Islands, 07122 Palma, Spain

**Keywords:** facial expression recognition, emotion recognition, UIBVFED, machine learning, convolutional neural networks, XAI, LIME, CEM

## Abstract

Recognizing facial expressions has been a persistent goal in the scientific community. Since the rise of artificial intelligence, convolutional neural networks (CNN) have become popular to recognize facial expressions, as images can be directly used as input. Current CNN models can achieve high recognition rates, but they give no clue about their reasoning process. Explainable artificial intelligence (XAI) has been developed as a means to help to interpret the results obtained by machine learning models. When dealing with images, one of the most-used XAI techniques is LIME. LIME highlights the areas of the image that contribute to a classification. As an alternative to LIME, the CEM method appeared, providing explanations in a way that is natural for human classification: besides highlighting what is sufficient to justify a classification, it also identifies what should be absent to maintain it and to distinguish it from another classification. This study presents the results of comparing LIME and CEM applied over complex images such as facial expression images. While CEM could be used to explain the results on images described with a reduced number of features, LIME would be the method of choice when dealing with images described with a huge number of features.

## 1. Introduction

To recognize facial expressions has been a persistent goal in the scientific image analysis community, as it is highly related to the observable representation of human emotions. Emotions are a means to understand human states through the huge amount of information they provide. Therefore, this information should be recognized to be used to improve the design of modern intelligent devices in order to increase their personalization and engagement.

Traditionally, since the rise of artificial intelligence, deep neural networks have the focus of the scientific community when dealing with the goal of recognizing facial expressions, specifically convolutional neural networks (CNN), as they allow images to be directly used as input. Although current CNN models can achieve high recognition rates, they give no clue about the nature of the process they follow to identify an expression. To gain confidence, there is an increasing need to be able to identify which factors are taken into account when a neural network outputs a result.

To try to explain the results obtained by intelligent systems has been a recurrent concern over the years. Late in the past century, symbolic reasoning systems such as MYCIN [1], GUIDON [2], SOPHIE [3], and PROTOS [4] were investigated to present, justify, and explain diagnostic reasoning. Developed in the early 1970s as a research prototype for diagnosing bacteremic bloodstream infections, MYCIN was able to describe hand-coded rules that aided in the diagnosis of specific cases. Research on intelligent learning systems has led to the development of systems that act as articulated experts, explaining problem-solving strategies to students. Some examples are SOPHIE, which was able to qualitatively justify solutions to electronic problems, and GUIDON, which added tutorial rules to complement the MYCIN domain-level rules to help explain medical diagnostic strategies.

Truth preservation systems were also developed to extend the capabilities of causal, rule-based, and logic-based inference systems, working with reasoning from conclusion to hypothesis by manipulating rules to provide an explanation. Such systems were able to generate explanations from inference traces. Expert clinical researchers created neural network-based decision supports for clinicians, attempting to develop dynamic explanations in which these techniques could increase the trustworthiness and credibility of patients.

As a result of these techniques and the rise of artificial intelligence in society, many academics and organizations began developing tools to help identify bias in systems. In 2010, due to the generalized use of artificial intelligence, great concerns arose about the search for a more transparent intelligence free of racial bias and gender discrimination. Marvin Minsky [5] addressed the need to use a humanistic intelligence for AI in order to achieve a much fairer intelligence.

Concerning neural networks, methods such as LRP [6] stand out. It searches an input vector for the characteristics that most influence the output of the neural network. Other well-known methods to explain the classification provided by a neural network are SHAP [7], permutation feature importance [8], ALE [9], GIRP [10], LIME [11], CEM [12] and anchor [13] methods.

All of these techniques have been referred to by the term XAI, which stands for explainable artificial intelligence [7,11]. XAI techniques have been introduced as a means to help to interpret the results and to rationally explain the decisions in order to evaluate the strengths and weaknesses of the model under study [14]. Of the aforementioned methods, SHAP uses the Shapley value to interpret the learning model and can be used on any predictive model. Its clear advantage is that it is straightforward to interpret, but in its pure form, it quickly becomes computationally expensive. Permutation feature importance, ALE and GIRP can only be applied globally, and cannot be used to explain specific predictions. Anchor has the drawback that when the number of feature predicates increases, the ability to explain more observations (coverage) highly decreases.

In the case of models dealing with images, one of the most-used XAI techniques in the human-computer interaction field is LIME [15]. LIME highlights the areas of the image that contribute to a classification. Recently, as an alternative to LIME, the contrastive explanation method (CEM) appeared, providing explanations in a way that is natural for human classification: besides highlighting what is sufficient to justify a classification, it also identifies what should be absent to maintain it and to distinguish it from another one. Therefore, in this work, we will focus on the analysis of LIME and CEM.

Although there have been attempts in the recent literature to compare the explanations obtained from different systems [16] and in different fields, such as in the context of decision support in diabetes self-management [17], we think that a close comparison of these two methods when working with complex images such as facial expressions could be useful for the scientific community as an aid to choose between these two XAI techniques in similar scenarios.

We first train a simple CNN model that recognizes emotions from images of facial expressions, and then we try to understand the behavior of our black-box model applying LIME and CEM over some of the predictions obtained. The main goal of this work is comparing the explanations provided by the two XAI techniques in the case study of images described with a huge number of features. Therefore, as a final step, we analyze and discuss the results regarding the pros and cons of each of these XAI methods when dealing with complex images such as facial expressions. The contribution of this work is not the trained CNN model but the understanding off how the two XAI techniques’ explanations contribute to the human comprehension of the generated predictions.

## 2. Materials and Methods

This section contains detailed description of data, data pre-processing, and the procedure followed.

### 2.1. Data Description: The UIBVFED Dataset

Our CNN model was trained on the UIBVFED dataset [18]. UIBVFED is a database made up of 20 synthetic avatars (10 men and 10 women, aged between 20 and 80, from different ethnicities) performing 32 facial expressions. The expressions are classified based on the six universal emotions according to the Gary Faigin classification (anger, disgust, fear, joy, sadness, and surprise) [19], plus a neutral emotion (see Table 1). The dataset is composed of 660 facial images from 20 virtual characters, each creating 32 facial expressions, plus a neutral expression. Table 2 shows the number of images per emotion.

The images of the UIBVFED dataset were generated according to the Facial Action Coding System (FACS) [20]. To generate the facial expressions, the deformations applied to the avatars corresponded to the action units (AUs) associated with each expression (more detailed information can be found in Mascaró Oliver and Amengual Alcover [18]). Hence, it was assured that the automatic labelling of the images was objective.

Moreover, the use of synthetic datasets has proved to be a good substitution for real-image datasets, as they obtain recognition rates similar to the real ones [21,22].

### 2.2. The Convolutional Neural Network

**Figure 1:**
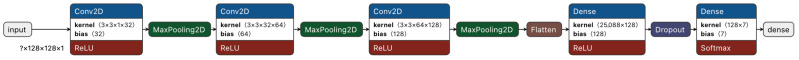
The convolutional neural network.

In this work, we used a convolutional neural network that obtains a grayscale image with a resolution of 128 × 128. The network applies three combinations of convolution, ReLu and max-pooling, and ends with two fully-connected layers. The convolutional layers extract characteristic treats of the image, the ReLu layer applies a max activation function, and the last fully connected layer computes the class scores resulting in one of our seven emotion classes. Zero padding and a stride of one pixel were used in the CNN. To reduce overfitting, we used a dropout layer between the two dense layers of our model. The CNN follows the scheme shown in Figure 1.

### 2.3. Data Pre-Processing

The pre-processing steps include the cropping of the face to reduce the influence of the background in the learning process, the conversion of the image to grayscale, and its resizing to fit the 128 × 128 pixel size of the input data in the CNN.

### 2.4. Procedure

After completing the pre-processing step over all the images of the UIBVFED dataset, we prepared the training and testing datasets. For the training dataset, we collected 80% of the data, and we took the remaining 20% for the testing dataset: to prevent information leakage, the dataset was split by selecting a random set of 16 avatars to construct the training dataset, leaving the remaining 4 characters for testing. Both datasets contained a class distribution that was representative of the complete UIBVFED dataset (see Table 3).

We trained the previously described CNN model with the training dataset. Then, the model was tested with the testing dataset, and the evaluation metrics in terms of global accuracy and a confusion matrix were computed.

As a final step, and to try to obtain an explanation of the model’s outcome, we applied two XAI approaches over the predictions: LIME and CEM.

### 2.5. XAI Approach

The output of a neural network is the result of applying complex mathematical decisions. This level of mathematical abstraction can diminish the user’s confidence in the decisions of a particular model. This means that although our model may obtain the correct decision, we cannot really assert with certainty its validity, as we have no clue of the process behind the model’s reasoning. Explainable artificial intelligence (XAI) tries to fulfil this gap, somehow interpreting the decision-making process of a given artificial intelligence (AI) model. Two of the more famous XAI techniques when dealing with images are LIME and CEM. We will use both of them to see how they perform in our recognition model.

#### 2.5.1. LIME

The local interpretable model-agnostic explanations (LIME) [11] is one of the most popular and commonly used XAI methods due to its simplicity and intuitiveness. It is agnostic because it can explain any model, treating it as a black box.

**Figure 2:**
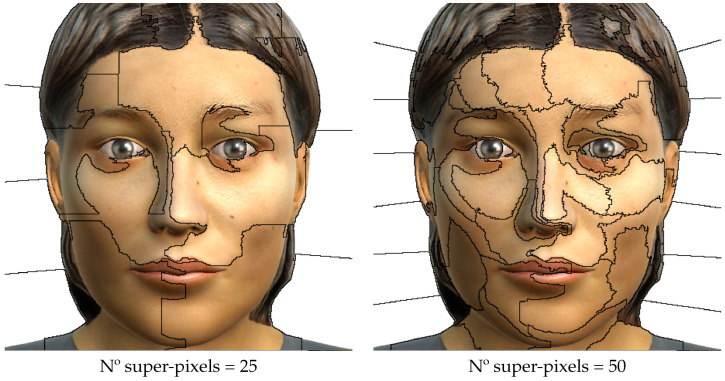
Example of the configuration of the number of super-pixels to segment into.

LIME depicts the main parts of the input that contribute to the prediction using a simple approach: it perturbs the inputs of the model and observes how the new predictions behave, and then, it learns how the model works using a linear model through the weighting of the perturbations. The obtained explanation is not globally valid, but it is accurate locally around the perturbed inputs. To explain an image classifier, LIME highlights the super-pixels, or collection of pixels that covers a connected area of the image (see Figure 2), which most justify the election of a given class. The super-pixels should correspond to specific patterns of the image, but normally, the user can only specify the resolution of the considered areas. This poses an additional difficulty, as significant features may lay in different super-pixels. However, we may have some kind of control if we know the relative size of the affected areas, as we can adjust the number of super-pixels and thus its size.

**Figure 3:**
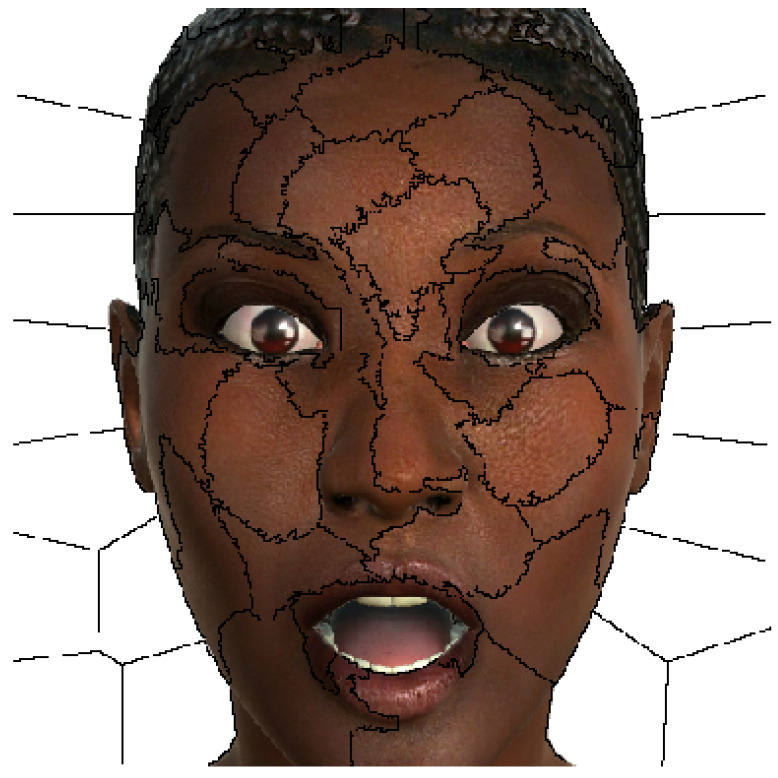
A facial expression image of the UIBVFED dataset showing the areas divided by super-pixels (n° super-pixels = 50).

In this study, we used the number of super-pixels of 50. In Figure 3, we can observed the areas in which our facial expression images are divided with that configuration of super-pixels.

#### 2.5.2. CEM

The contrastive explanation method (CEM) [12] is an XAI system that tries to go beyond the correlations of input variables with the output, as LIME does. It works with the minimum and sufficient characteristics capable of justifying a solution (referred to as pertinent positives or PP), and the minimum characteristics that can change the result if they are present in the image (referred to as the pertinent negatives or PN). This way of working by locating missing or present features is totally cognitive and very similar to how the human brain performs to recognize and identify objects.

**Figure 4:**
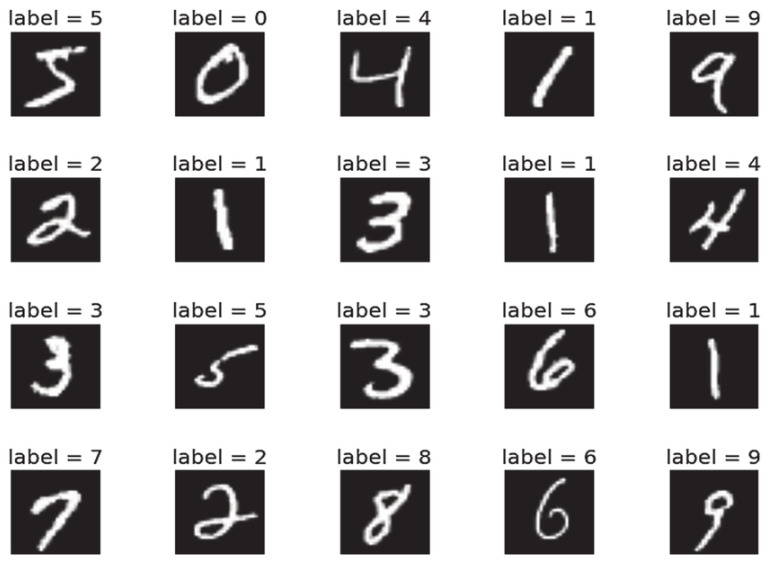
Sample images of the MNIST dataset. The MNIST dataset [23] is a large database of handwritten digits that is commonly used for training machine learning models, and it is composed of black and white images containing simple patterns.

**Figure 5:**
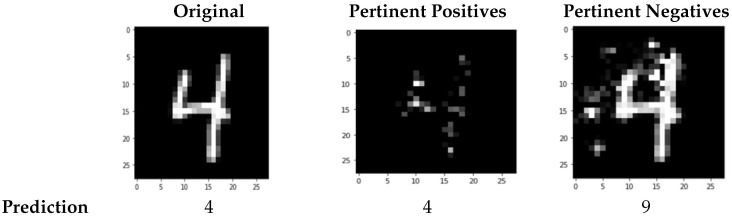
Results of applying CEM to one image of the number four in the MNIST dataset.

CEM has been traditionally validated in the literature for the MNIST dataset [12]. For instance, applying CEM to the MNIST dataset (see Figure 4), the explanations obtained for an image of the number four (see Figure 5) were shown in the pertinent positives for the characteristics that have to be present to compose the silhouette of the number four: one vertical small arm at the left side and one vertical long arm on the right side joined by a horizontal line. The pertinent negatives showed characteristics that, when added to the original image, would change the image to be the number 9: a path that joins the upper side of the left small arm with the upper side of the right long arm.

## 3. Results and Discussion

**Figure 6:**
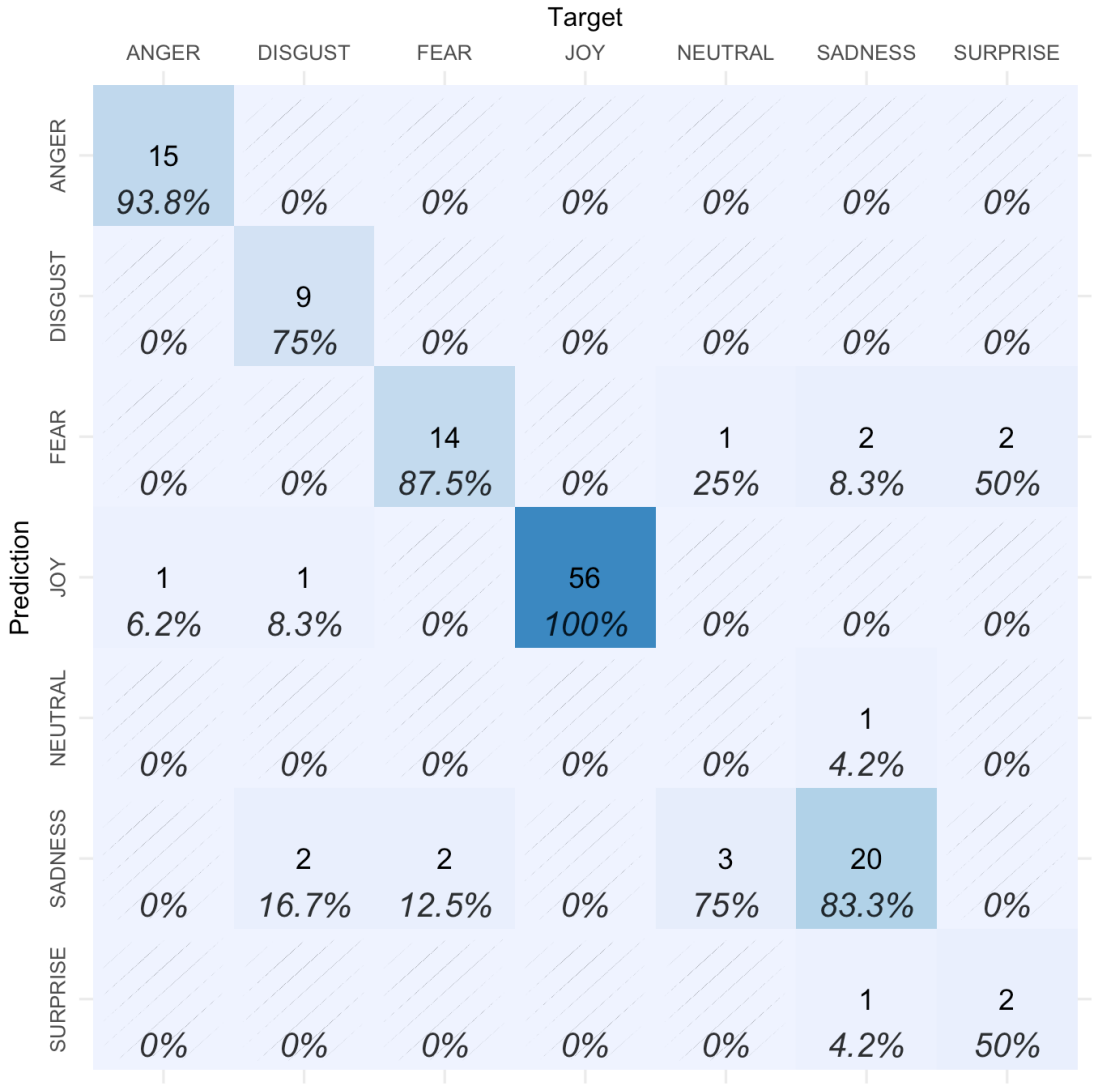
Confusion matrix of the emotions’ classification performed by the CNN model (darker colors correspond to higher accuracy).

The convolutional neural network, when tested, had a global accuracy of 0.88, and the resulting class classification is enumerated in Figure 6. Joy, anger and fear obtained the best results (ranging from 88% to 100%), whereas the worst-identified emotion was neutral (0%). It is also interesting to review the information of the most misleading emotions: neutral was confused with sadness in 75% of the images, and surprise was confused with fear in 50% of the cases.

### 3.1. Understanding the Behavior of the Model with LIME

To try to understand the behaviour of the model, we first applied LIME to obtain an explanation for some of the predictions generated by the model.

**Figure 7:**
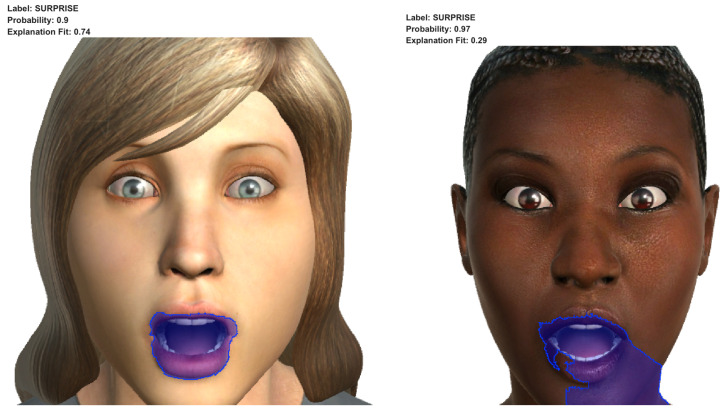
Results of applying LIME to the images of surprise correctly labelled as the emotion of surprise.

In Figure 7, the results of applying LIME to the predictions of the two images correctly labelled as the surprised emotion can be observed. In both cases, the super-pixels highlighted of the images coincide with the area of the mouth, and they depicted an open mouth with an oval shape and without any muscle tension (corresponding to the action units AU25—lips part and AU26—jaw drop).

In accordance with Faigin [19], facial expression recognition depends on the role of the face muscles. In his work, the author focuses on the action of muscles in three key areas of the face: (1) the forehead and brows, (2) the eyes, and (3) the mouth and chin. The facial features of the mouth highlighted in Figure 7 are in line with the facial features of the facial expression associated with the emotion of surprise in the Gary Faigin work (see first row in Table 4).

**Figure 8:**
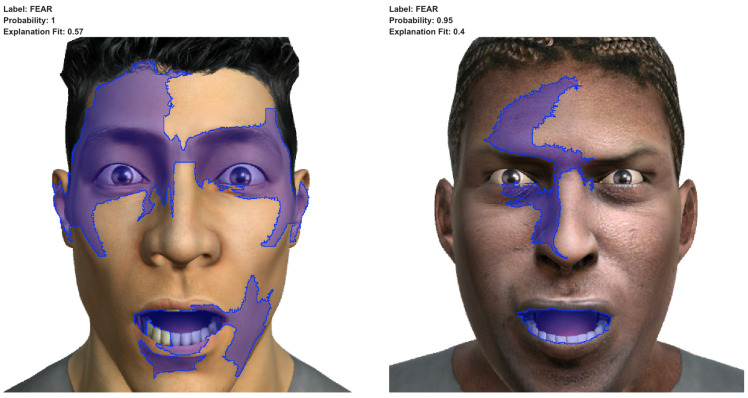
Results of applying LIME to images of fear correctly labelled as the emotion of fear. The left one corresponds to the facial expression *very frightened*, and the right one corresponds to the facial expression *terror*.

In Figure 8, we can observe the results of applying LIME to the predictions of two images correctly labelled as the fear emotion. In both cases, the super-pixels highlighted on the images correspond to the areas of the mouth and the eyebrows. They depict an open and widened mouth (corresponding to the action units AU25—lips part and AU26—jaw drop) with the eyebrows lifted straight up and pulled closed together (corresponding to the action units AU1—inner brow raiser and AU2—outer brow raiser in the left image and to the action unit AU2—outer brow raiser in the right image). In the left image (corresponding to the facial expression *very frightened*), we can see the super-pixels highlighted that correspond to the action unit AU5—upper lid raiser, and, in the right image (corresponding to the facial expression *terror*), we can see the super-pixels highlighted that correspond to the action units AU6—cheek raiser and AU7—lid tightener. All of these facial features are in line with the facial features expected for those facial expressions in the Gary Faigin work (see the second and third rows in Table 4).

**Figure 9:**
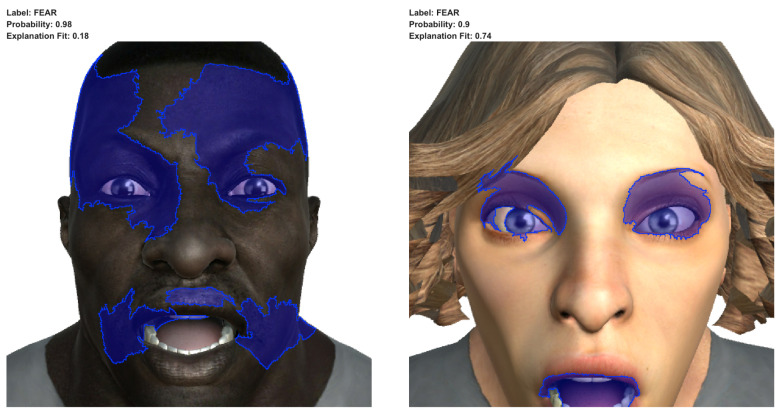
Results of applying LIME to the images of surprise incorrectly labelled as the emotion of fear.

In Figure 9, we can observe the results of applying LIME to the predictions of two images of the facial expression associated with the emotion of surprise incorrectly labelled as fear. As expected, the super-pixels highlighted in the images correspond to the areas of the mouth and the eyes and eyebrows. They depicted an open mouth (corresponding to the action units AU25—lips part and AU26—jaw drop) and open eyes with the eyebrows straight up (corresponding to the action units AU1—inner brow raiser, AU2—outer brow raiser and AU5—upper lid raiser. These facial features corresponding to the facial expression associated with the emotion of surprise (the correct emotion) are also present in the facial expressions of terror and very frightened, associated with the emotion of fear (the incorrect prediction returned by the model). Hence, according to the explanation provided by LIME, the results returned by the model could be coherent with the information that it extracted from the images.

These last explanations are also coherent with Gary Faigin’s work: in accordance with Faigin [19], there are facial expressions that share facial features or very similar facial features; therefore, there exist facial expressions that could be easily misled, and consequently, their associated emotions could also be misled. Table 5 details the similar facial expressions (and, consequently, the similar emotions associated) to the emotions of surprise and fear, according to their facial features.

As a summary of the application of LIME over the predictions of our model to classify facial expression images into emotions, observing the explanations provided by LIME, it is feasible to understand why the model returns a prediction. Specifically, it can be inferred that the model focuses on the regions of the images corresponding to the areas where the facial features of the facial expressions occur to classify an image: the areas of the mouth, eyes and eyebrows. Furthermore, we have also been able to prove that the behaviour of our model is coherent with the theory of Faigin with the correct classifications and also with the misled classifications.

### 3.2. Understanding the Behavior of the Model with CEM

**Figure 10:**
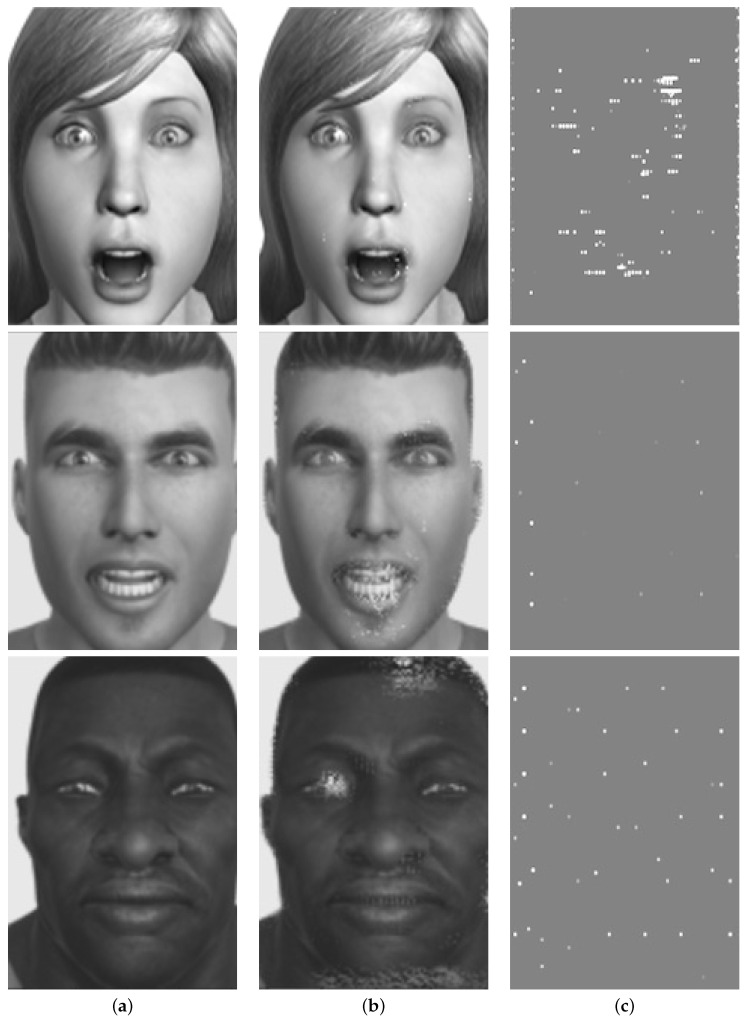
Results of applying CEM to a grayscale input image (**a**) showing the (**b**) explanation of the pertinent negatives and the (**c**) explanation of the pertinent positives.

When we apply the contrastive explanation method (CEM) to the same dataset using as input a grayscale image (see Figure 10a), we obtain as output the images corresponding to both the pertinent positives, i.e., the minimal set of features that lead to a given prediction P (see Figure 10c), and the pertinent negatives, i.e., the minimal set of features that should be absent to maintain a decision P instead of changing the decision to a closest class Q (see Figure 10b).

The first row in Figure 10 shows the explanation obtained by the CEM method with an input image corresponding to a surprise emotion, predicted as surprise by pertinent positives and as fear by pertinent negatives. The second row in Figure 10 shows the explanation obtained with an input image corresponding to the emotion of anger, predicted as anger by pertinent positives and as sadness by pertinent negatives. Finally, the third row in Figure 10 shows the explanation for an input image corresponding to sadness, predicted as sadness by pertinent positives and as fear by pertinent negatives. As we can see, in all the results, the pertinent negatives somehow show areas that could help in understanding the prediction, but they are not even close to how the LIME method shows it. The pertinent positives, composed mainly of isolated pixels, give no clear clues regarding the explanation.

In the generated explanations with CEM, we can see that the results, unlike what happens in LIME, are not very intuitive. The pertinent negatives might give us insight into which areas of the input image are most influential to induce change in the classification of the input image, but the explanations obtained through the pertinent positives are difficult to interpret as they are related to the pixel level, and no homogeneous connected areas can be really perceived. LIME uses super-pixels (areas of connected pixels) to divide the original image and extracts its conclusions based on how these super-pixels influence the results. However, CEM uses individual pixels that do not need to be related to a specific region of the image, and this does not help human comprehension.

## 4. Conclusions

The recognition of facial expressions is a topic frequently addressed in the field of human–computer interactions since the emotions conveyed through expressions provide a huge amount of information. Traditionally, convolutional neural networks (CNN) have been one of the most-used computational learning systems to recognize and analyze human expression, as images can be directly used as input to the training process. Although current CNN models can achieve high recognition rates, as black-box models, they give no clue regarding the nature of the process they follow to identify an expression, leading to trust issues on the predictions returned by the models. Explainable artificial intelligence (XAI) has been developed as a means to help interpret the results obtained by models.

In this work, we have compared LIME and CEM to provide explanations for the case study of images of facial expressions. For this purpose, we have used the UIBVFED dataset, which contains facial expressions of virtual avatars of different ages, sexes and ethnicities.

Results show evidence that, in this case study, while LIME generates explanations that can be easily understandable by human beings and that follow the guidelines extracted from the Faigin studies, CEM does not provide satisfactory answers in this regard. Probably, the huge amount of characteristics present in the images limit the effectiveness of CEM, which shows much more convincing results with images of lower complexity, such as the images of the MNIST dataset. In fact, in the examples that we have used, the explanations resulting from the CEM method do not facilitate human understanding.

A plausible explanation would be that whilst LIME uses super-pixels to divide the original image and extracts its conclusions based on how these super-pixels influence the results, CEM uses individual pixels that do not need to be related to a specific region of the image, and this does not help human comprehension.

To conclude, in a scenario where we must choose between LIME and CEM, we propose the use of LIME to understand the predictions in the case of dealing with complex images (with huge amount of characteristics) such as facial expression images, as the organization of the image in super-pixels could assist the inference of meaning to the clustered features. On the other hand, CEM could be used to explain the results on images that can be described with a reduced number of features, which also provide valuable information through the pertinent negatives.

## Figures and Tables

**Table 1:** Emotions associated with each facial expression according to Gary Faigin classification.

Emotion	Facial Expression
Neutral	Neutral
Anger	Enraged compressed lips, enraged shouting, mad, sternness, anger
Disgust	Disdain, disgust, physical repulsion
Fear	Afraid, terror, very frightened, worried
Joy	False laughter 1, false smile, smiling closed mouth, smiling open-mouthed, stifled smile, laughter, uproarious laughter, false laughter 2, abashed smile, eager smile, ingratiating smile, sly smile, melancholy smile, debauched smile
Sadness	Crying closed mouth, crying open-mouthed, miserable, nearly crying, sad, suppressed sadness
Surprise	Surprise

**Table 2:** Number of images per emotion of UIBVFED database.

	Anger	Disgust	Fear	Joy	Neutral	Sadness	Surprise
N° of images	80	60	80	280	20	120	20

**Table 3:** Class distribution of the UIBVFED, training and testing datasets.

Dataset	Anger	Disgust	Fear	Joy	Neutral	Sadness	Surprise	Total
UIBVFED	80	60	80	280	20	120	20	660
Training	64	48	64	224	16	96	16	528
Testing	16	12	16	56	4	24	4	132
Proportion	0.12	0.09	0.12	0.42	0.03	0.18	0.03	1

**Table 4:** Summary of the main facial features of the facial expressions associated with the emotions of suprise and fear extracted from Faigin conclusions (more detailed information can be found in Faigin [19]) and their corresponding action units (AUs).

Emotion	Facial Expression	Eyebrows	Action Units	Eye	Action Units	Mouth	Action Units
Surprise	Surprise	Highly raised or relaxed	AU1—inner brow raiser, AU2—outer brow raiser	Widely opened with lower lid relaxed	AU5—upper lid raiser	Dropped open. Oval in shape	AU25—lips part, AU26—jaw drop
Fear	Terror	Lifted up, more straight than arched	AU1—inner brow raiser, AU2—outer brow raiser	Widely opened with raised lower lid	AU5—upper lid raiser, AU6—cheek raiser, AU7—lid tightener	Opened and widened	AU11—nasolabial deepener, AU25—lips part, AU26—jaw drop
Fear	Very frightened	Lifted up, more straight than arched	AU1—inner brow raiser, AU2—outer brow raiser	Very widely opened	AU5—upper lid raiser	Opened and widened	AU11—nasolabial deepener, AU25—lips part, AU26—jaw drop
Fear	Afraid	Lifted up, more straight than arched	AU1—inner brow raiser, AU2—outer brow raiser	Not opened much wider than usual	AU5—upper lid raiser	Slightly dropped open	AU22—lip funneler
Fear	Worried	Lifted up, more straight than arched	AU1—inner brow raiser, AU2—outer brow raiser	Not widened	AU5—upper lid raiser	Squeezed tight	AU15—lip corner depressor, AU17—chin raiser, AU18—lip puckerer

**Table 5:** Similar facial expressions associated with the emotions of surprise and fear, according to their facial features (more detailed information can be found in Faigin [19]).

Emotion	Facial Expression	Similar to
Surprise	Surprise	Fear
Fear	Terror	Surprise
Fear	Very frightened	Surprise
Fear	Afraid	Sadness
Fear	Worried	Suppressed sadness

## Data Availability

Publicly available datasets were analyzed in this study. These data can be found here: http://ugivia.uib.es/uibvfed/ (accessed on 17 October 2022).
